# An analysis of E-governance in Pakistan from the lens of the Chinese governance model

**DOI:** 10.1016/j.heliyon.2024.e27003

**Published:** 2024-02-27

**Authors:** Muhammad Atique, Su Su Htay, Muhammad Mumtaz, Naqib Ullah Khan, Ali Altalbe

**Affiliations:** a Digital Media Department, Stirling College, Chengdu University, China; b College of Public Administration, Huazhong University of Science and Technology, Wuhan, China; c Department of Public Administration, Fatima Jinnah Women University, Rawalpindi, Pakistan; d School of Public Administration, Central South University, Yuelu District, Changsha, Hunan, 410017, China; e Department of Computer Science, Prince Sattam Bin Abdulaziz University, Al-Kharj, 11942, Saudi Arabia

**Keywords:** E-governance, Digitalization, Good governance, Governance transformation, ICTs, China & Pakistan

## Abstract

In this era of digitalization, the role of information and communication technology (ICT) has significantly increased. The integration of ICT into the government system has improved efficiency and working processes. Some countries such as China have successfully integrated ICT into their governance system. However, many other countries especially the developing world are yet to effectively utilize the role of ICT in their governance structure and these countries are struggling to produce a better governance system. It is, therefore, imperative for the developing world to learn from successful nations and devise their governance systems so that ICT can be fully utilized and produce good governance. However, such comparative analyses are not done as such to bring strengths and weaknesses in the integration of ICT into the governance system especially in developing countries' governance systems. This study contributes by conducting a comparative study on the China-Pakistan e-government progress. China has widely implemented e-government, which has helped the country to ensure good governance. Pakistan, on the other hand, is also moving towards digitalization and making efforts to implement e-government. This research examines the United Nations' E-Government Development Index (EGDI) reports and rankings. The findings of our research show that China has significantly improved its ranking, whereas Pakistan's ranking has indicated a gradual decline except for the year 2008. This happened because of a lack of investment in infrastructure, scarcity of financial resources, weak institutional capabilities, and limited access to advanced technologies. Moreover, there is a big gap between public policy and public implementation in Pakistani scenarios. However, it has been dug out in this study that employing the Chinese model and seeking cooperation with China can improve e-governance ranking and overall governance in Pakistan. The study advances the understanding of e-governance and its challenges in Pakistan and the findings of the study will assist researchers, policymakers, and officials in the implementation and development of e-projects in Pakistan.

## 1 Introduction

The integration of ICTs in the government system has become a prominent feature of the government system in today's digitalized world [1]. E-governance has emerged as an effective tool in reducing corruption, enhancing transparency, increasing convenience, and bolstering GDP growth [2,3]. E-governance enhances citizens' participation directly in their country's governance, reduces overall costs, and widens the government's reach. Moreover, E-government promotes effectiveness in the government system by improving the quality of information and services provided to the public by utilizing ICT in a simple, cost-effective, and efficient manner [4].

The various e-governance models demonstrate that e-government has the potential to stimulate economic growth and economic empowerment [5]. E-governance reinforces and plays a pivotal role in economic growth and economic stability in any country [6]. However, there is a noticeable disparity in how developing and developed countries implement e-government. The success and progress of underdeveloped countries have been significantly influenced by the implementation of e-government [7].

The integration of ICTs in national development strategies has been identified as a positive step in utilizing technology to drive progress and meet development goals [8]. The effective employment of ICTs can improve governance, bolster economic growth, and improve living standards [9]. The impact of investment in ICTs is highly reliant on a variety of factors including the level of infrastructure development, government policies, regulations, and the level of technology adoption by people [10]. In some cases, ICTs have not lived up to expectations and outcomes have been mixed. As a result, attention has shifted towards the use of ICTs as a tool to support good governance and holistic development, rather than solely focusing on the technological aspect [11]. Countries across the world are exercising ICT practices to improve their governance systems.

Pakistan has taken many steps to implement effective e-governance in the country to facilitate the public in the digitalized world [12]. During the COVID-19 pandemic, Pakistan was compelled to adopt digital services and ensure transparency and ease in the governance process [13]. Since then, the country has been moving towards digitalization. However, Pakistan has yet to fully integrate ICT into its existing system. Pakistan's ranking on the e-governance Index, E-participation Index, and Human Development Index has seen no significant improvement as compared to other countries in the region [13]. E-governance is key to good governance. However, implementing and integrating E-governance without an effective model or suitable framework is challenging. Considering the historical records and achievements of China can be taken as a suitable model for e-governance.

China has produced significant achievements in E-governance and it has made substantial achievements through bringing ICT into the governance system [14]. China has emerged as a leader in E-governance. On the other hand, Pakistan is struggling to effectively incorporate E-governance into its governance system. However, Pakistan is on the way to learning from China how to improve its system. It is therefore needed to investigate why Pakistan is unable to integrate ICT into its governance system.

This study is conducted to make a comparative analysis of China and Pakistan to identify key challenges of E-governance for Pakistan in light Chinese model of e-governance. The objective of this research is to analyze China and Pakistan's e-governance and explore the differences in e-governance policies between China and Pakistan. The study also identifies key challenges for e-governance in the Pakistani context by looking at the case of China. Additionally, the study brings key lessons from the Chinese e-governance model for other countries to learn. The finding of this study will also contribute to adopting effective e-governance policies and ensuring good governance in the different countries especially Pakistan by integrating ICTs into the existing system. This research is comprised of five sections. The first section deals with the introduction. The second section is a literature review which provides an understanding of the existing studies and finds the research gap. The third section presents the case study, datasets, and methodology used in the research. The fourth section discusses and analyzes the research findings, comparing and contrasting China and Pakistan. Finally, the study concludes with recommendations and policy implications.

## 2 Literature review

### 2.1 The concept of E-governance

The concept of the integration of ICTs in governance has existed for a long period in human history. The ICTs are a relative innovation, but their incorporation into the governance system has existed long [15]. Each generation has seen unique technological development in response to new trends in societal, economic, and political spheres [16]. The utilization of information and communication technologies (ICTs) in the governance system to improve efficiency in the process is called e-governance [17]. E-governance involves the incorporation of ICTs both in terms of devices and methods, into the operations of government departments [18]. This trend sometimes, also called digitalization means the increasing role of data in various social processes, including those in public service agencies [19]. The main goal behind adopting an e-governance system is to minimize corruption through reduced human involvement, increasing productivity, and providing citizens with efficient, fair, transparent, and accountable access to services and goods [20]. These aims are in line with the principles of good governance, including accountability, anti-corruption measures, adherence to the rule of law, and overall government efficiency [21,22]. The concept of E-governance and utilization of ICT is being considered in many countries. However, in many countries, it is yet to fully integrate E-governance and utilize its effective benefits. The new generation has witnessed increased attention given to the utilization of ICTs. Siar (2005) has highlighted six broader functions of e-governance [23]. His findings show that e-governance is helpful in citizens' knowledge of their community, enhancing the accessibility of government services, and improving transparency and accountability in the governance system. Besides, e-governance has the potential to develop an understanding of the people in the policy-making process enhance their participation in decision-making, and facilitate communication collaboration between government and people, government-to-government, civil society, and other entities. ICTs are important in administrative services for public service delivery. ICTs play a critical role in increasing the efficiency of the administrative system by creating better communication between citizens and the government [24]. ICTs can enhance the governance process by creating channels of communication and facilitating an efficient public discourse [24], p. 4). In addition, Banerjee et al. (2020) reported that the integration of ICTs into government practices helps in reducing corruption and enhancing government accountability [25].

In 2018, the United Nations E-government survey revealed that e-government functions are beneficial for achieving sustainable development goals 2030 agenda, considering it as a significant mechanism for promoting sustainability. In addition, it says ICTs are a significant driver of resilience and sustainable development stems from the open government approach United Nations, 2018 [26]. ICTs help promote effectiveness in service delivery to citizens and businesses. It improves the quality of government services enhances public participation in the governing process [27,28], and enhances public service efficiency in terms of reduction in corruption and promoting informed decisions.

The utilization of ICTs in the administrative field can help in reducing corruption [29]. ICTs improve transparency and accountability. ICTs increase the access of citizens to government information and enhance their role in decision-making and service delivery [30]. Studies of digitization in procurement processes, such as the OPEN system in the Seoul Metropolitan Government (SMG) and the Government e-Procurement System (GPS), showed that digitization leads to reduced corruption due to increased transparency [31]. Similar results were seen in studies of procurement processes in India [32]. By making procurement information easily available and accessible to the public, digitization enhances trust and satisfaction in government. A study by Akpan (2017) shows that automation of financial and procurement processes has helped in saving the resources of the government which has helped in increasing revenue [33]. However, the utilization of all these aspects through ICTs is not fully integrated with many countries, especially in the developing world.

Furthermore, studies by Bartenberger & Grubmüller (2014) and Kneuer & Harnisch (2016) have reported that through ICTs collaborative and participatory governance can be ensured [34,35]. Oseni et al. (2015) believe that ICTs increase employment and enhance public safety [36]. Ansari (2015) and Masiero (2015) have identified that the role of ICTs is crucial in improving food security [37,38]. Its role is crucial in promoting fair land administration [39]. Xia (2017) and Din et al. (2017) have examined the role of ICTs in the governance system for promoting political modernization and inclusion [40]. It has been seen that the wide-ranging impact of ICTs on various aspects of human life, reinforces the idea that integrating ICTs into government processes can help achieve good governance. According to Kneuer & Harnisch (2016), e-government and e-participation have emerged as critical tools of public administration and political interaction for the last three decades [35].

Moreover, some other studies have also concluded that the use of ICTs in the governance system can improve efficiency in the delivery of public services. A study by Bertot et al. (2012) and Rubasundram & Rasiah (2019) concludes that the automation process helps in reducing the need for human intervention and decreasing the chances of errors [41]. For example, the integration of technology in the financial processes of the Edo State government in Nigeria increased the speed of payroll processing and ensured prompt payment of salaries to public servants. Sharmi & Islam (2013) found that e-governance in Bangladesh has been observed to have a positive impact on transparency, accountability, and consistency in government processes, thereby reducing the likelihood of corruption and other unethical practices [42]. Neumann et al. (2012) report that the lack of transparency in government processes can lead to an environment where corruption becomes more attractive and incentivizing good behavior becomes difficult [32]. Thus, e-governance plays an important role in promoting good governance by ensuring transparency, accountability, and efficiency in government processes. However, bringing these aspects into governance systems is complicated and demands further investigations and a way forward.

Ahmed et al. (2017) have analyzed the e-governance initiatives in Pakistan, including the National Portal, the Citizen Feedback Monitoring System, and the e-procurement System [43]. The study found that these initiatives have improved transparency and accountability in government processes, as well as increased access to information and services for citizens. Ahmad et al. (2017) assessed the impact of e-governance on citizen satisfaction with government services in Pakistan where it was found that e-governance has improved the quality and accessibility of government services, leading to increased satisfaction among citizens [43]. However, several challenges to the implementation of e-governance in Pakistan, including a lack of infrastructure, limited awareness among citizens, and insufficient investment in ICTs have been reported. Similarly, Nishat (2021) explored the challenges and opportunities for e-governance in Pakistan, with a focus on the role of public-private partnerships [44]. The study found that partnerships between government and private sector organizations can play a key role in addressing the challenges and improving the effectiveness of e-governance initiatives in Pakistan. However, there is a need to investigate how and with what conditions such partnerships and coordination among various institutions can be ensured to address the challenges of e-governance in Pakistan.

### 2.2 E-governance in China

The beginning of the twenty-first century saw remarkable initiatives by the Chinese government to improve governance through e-governance [45]. The Chinese government, in 2001, announced to achieve the goal of ensuring 80% of urban government agencies' facilities operated online by 2005 [46]. Since then, China has been at the forefront of e-governance development and implementation for many years. The Chinese government has recognized the importance of information and communication technologies (ICTs) in improving the efficiency and transparency of government operations and delivering public services to citizens [13]. In 2003, the Chinese government launched the “Government Online” project, which aimed to provide online services to citizens and businesses and enhance transparency in government operations [47]. Over time, China has been engaged in taking numerous initiatives to improve its governance systems.

In 2006, the government launched the “Public Information Online” project, which aimed to make government information more accessible to the public through digital channels. In 2008, the government launched the “Digital China” initiative, which aimed to promote the development of ICTs in various sectors, including e-governance [48]. Similarly, in 2015, the government launched the “Internet Plus” initiative, which aimed to integrate Internet technologies with traditional industries, including government services [49]. In 2016, the government launched the “Smart City” initiative, which aimed to use ICTs to improve urban governance and enhance the quality of life for citizens [50]. Moreover, in 2018, the government launched the “Xinjiang Cloud” initiative, which aimed to provide online services to citizens in the Xinjiang region and enhance social stability through e-governance [40]. China has been developing systematically in its e-governance over time to facilitate its citizens.

China has adopted a unique approach to providing government services to its citizens by leveraging social networking applications. While some countries are hesitant to outsource government services to private corporations and share personal data, China's focus is on ensuring its citizens use these services [51]. The UN's 2018 study on e-government indicates that mobile technology has the most significant potential for expanding internet usage, and China has been driving this expansion with its strong mobile internet infrastructure. E-governance has emerged as key for efficient public service delivery.

E-governance has led to increased efficiency for both government agents and citizens in China by bringing various online systems. Online transactions have replaced paperwork, resulting in cost savings for both the government and citizens [52]. As a result, citizens can interact with the government at any time, save time, and avoid the inconvenience of traveling to physical government agencies. E-governance is available at all levels of government in China, with official websites accessible in over 58,000 locations [53]. In 2015, e-governance covered 99.1% of city-level, 100% of national and provisional-level, and over 85% of county-level governments [13]. Local governments in China have also increased the use of mobile apps to provide more convenient services to citizens. Additionally, big data has been utilized by various city governments to inform policy-making and further develop e-governance [54]. China's development in e-governance and good governance has set an example for other countries, especially for developing and emerging economies to follow China's model.

### 2.3 E-governance in Pakistan

Like any other country, Pakistan also recognizes the importance of e-governance. The concept of e-governance in Pakistan can be traced back to the early 2000s when the government recognized the need to adopt modern technologies to enhance the efficiency and transparency of its public services [55]. In 2002, the Government of Pakistan launched its first e-government directorate which aimed to provide a platform for citizens to communicate their complaints and suggestions to government officials. In 2003, the National Database and Registration Authority (NADRA) introduced an electronic ID card system that enabled citizens to obtain secure and computerized national identity cards [56]. Similarly, in 2004, the government launched the “e-Police” project in Islamabad to digitize police records and improve the efficiency of law enforcement agencies while in 2005, the Punjab Information Technology Board (PITB) was established to promote and implement e-governance initiatives in the province of Punjab [57]. Pakistan has been also taking steps to promote e-governance in the country at national and subnational/provincial levels.

In 2010, the Sindh government launched the “Sindh Online” project, which aimed to provide online services to citizens, including birth and death certificates, property registration, and driving licenses. In 2012, the Khyber Pakhtunkhwa government launched the “e-Khidmat” project, which aimed to provide a range of citizen-centric services, such as passport applications, driving licenses, and property registration [58,59]. In 2017, the government launched the “Pakistan Online Visa System,” which enabled foreign tourists and businessmen to apply for visas online [60]. In 2018, the federal government launched the “Ehsaas” program, which aimed to provide financial assistance to the poor and vulnerable segments of society through a digital payment system [61,62].

In recent years, the number of internet users has seen a sharp rise, reflecting the country's technological ambitions. Though Pakistan is a developing nation, people demand e-services from the government for facilitation [63]. The Citizen Portal launched by the government of Pakistan in 2018 is an important initiative that has transformed the way citizens interact with the government [62]. It is an online platform that enables citizens to lodge complaints and provide feedback on government services, which can then be addressed by relevant. In 2018, Pakistan released a plan to increase the number of digital transaction accounts to increase the number of digital transaction accounts from 10 million to 50 million by 2023 [55]. This would be achieved through various initiatives, such as the introduction of low-cost mobile wallets and the promotion of agent banking. The plan aimed to increase the share of digital payments in overall transactions from 2% to 45% by 2023 [64]. This would be achieved by creating awareness among the general public about the benefits of digital payments and introducing incentives for merchants to accept digital payments [65]. Various crises such as COVID-19 have reinforced the promotion of digitalization in the country.

The COVID-19 pandemic has played a significant role in accelerating the adoption of e-governance systems in Pakistan [66]. The pandemic created a sudden need for remote access to government services and digital platforms, which has led to an increased focus on e-governance and digital transformation in the country. In response to the pandemic, the government of Pakistan has taken several initiatives to promote e-governance and ensure the provision of essential public services through digital channels. For instance, the National Command and Operation Center (NCOC) set up a digital dashboard to track the spread of COVID-19 across the country and provide real-time data and information to policymakers and the public [67]. Similarly, the government has launched several digital platforms to facilitate the provision of health services, such as the “Corona Helpline” and “Sehat Tahaffuz” apps, which enable citizens to access information and medical advice related to COVID-19. Moreover, the government has also implemented several measures to support remote learning, such as the “TeleSchool” and “e-Taleem” programs, which provide digital educational content to students across the country [68]. Despite these initiatives, Pakistan is still far from fully utilizing e-governance. Therefore, Pakistan needs to learn from others, especially from China which has set a successful example to follow for the development world to ensure effective governance in the turbulence or crises time.

## 3 Methodology

A comparative analysis of China and Pakistan has been made based on the sustainable indicators from 2005 to 2022. For this comparative study on China and Pakistan, data has been collected through the UN 2022 E-government survey report indicators which include the E-government Development Index (EDGI) ranking, the Human Capital Index (HCI), the Online Service Index (OSI), and the Telecommunication Infrastructure Index (TII), and E-Participation Index (EPI). The EGDI evaluates the e-government plans, strategies, and national websites. Also, it makes a comparative measurement of the countries' e-government work relative to one another and is a weighted average of normalized scores of the three components (OSI, TII, and HCI). For equal variance, z-score standardization is executed for each component indicator, with the EGDI showing considerable dispersion without it. Socio-economic data has been gathered from the World Bank development indicators, including the Human Development Index (HDI), real GDP growth, the Globalization Index, and ICT exports.

## 4 Analysis and discussion China-Pakistan E-government comparison

### 4.1 Pakistan E-governance initiatives in recent years

The COVID-19 pandemic accelerated the need for e-services and the public service sector in Pakistan. The government has been utilizing Information and Communication Technology (ICT) to promote digitalization, infrastructure development, innovation, human resource development, legislation, women's development, local hardware manufacturing, research and development, software exports, software technology parks, and e-governance. These efforts are aimed at ensuring that Pakistan remains at the forefront of technology and digitalization [69].

Pakistan's IT sector has been growing steadily, with 1500 registered IT companies in the country as of 2018. The industry is supported by a strong telecom sector and a skilled workforce of 110,000 English-speaking IT professionals, with 24,000 engaged in exports. In 2019, the government focused on its e-commerce policy, emphasizing regulatory facilitation, financial inclusion, empowerment of youth and SMEs, taxation, consumer protection, data ownership, and data localization [70]. Public Policies and implementation frameworks are essential for practically executing the decisions related to any reform or new advancement.

The government has also been working on progressive policies. Many policies such as the National Broadband Policy 2021, National Cyber Security Policy 2021, and National Freelancing Facilitation Policy have been developed in recent years to benefit the common people. Despite various policy initiatives and positive developments, Pakistan has yet to fully benefit from the ICTs in its system.

Since 2018, Pakistan has been making efforts to bring improvement in its e-government ranking. Though it has taken significant measures, remains far behind compared to China made significant progress (UN report 2018 and GOP 2017 report). In October 2018, a complaint website was launched (www.pmo.gov.pk/) at the federal, and provincial, as well as for overseas Pakistanis to ensure effectiveness in the country's e-governance system (PMO, 2020). Moreover, visa, passport, and national identity card services (www.nadra.gov.pk) are now online which is considered are significant initiative for providing easier access to public services.

The Digital Pakistan Policy 2021 is an important step towards transforming Pakistan into a digitally developed country. The policy covers various aspects of e-commerce and ICT, including regulation, financial inclusion, youth empowerment, consumer protection, and taxation, infrastructure, and data sovereignty. The advancements in ICT technology and the Fourth Industrial Revolution have provided Pakistan with the opportunity to become more connected and to enhance the sharing of information and data through web portals. The implementation of the Digital Pakistan Policy will support the country's efforts to achieve sustainable socio-economic development. However, Pakistan's record for implementing public policies is not up to the mark [71], so it is important to learn implementation actions from the Chinese case study model.

### 4.2 Comparison and discussion on E-governance progress

China and Pakistan have both been making efforts to improve their e-governance systems in recent years. However, there are some notable differences in their progress and development. China has made significant strides in e-governance over the past few decades, with improvements in infrastructure, management, and participation. China has seen steady growth in its e-government ranking and mean download speeds and has implemented various initiatives to improve online services, human development, and telecommunication infrastructure. As a result, China's e-governance model has been recognized as one of the most advanced in the world.

In contrast, Pakistan's e-governance development has not been as effective as China's [72]. The indicators for the efficiency of E-governance are quite low in the case of Pakistan. While the country has made some positive initiatives to improve e-governance, its e-government ranking has been lower than China's and has declined over the years [66,73]. Pakistan should adopt and implement an effective e-governance model for better management, facilitation, and participation. In addition, it should ensure better online services, and promote human development to contribute to the development of the country by promoting e-governance for good governance.

**Fig. 1:**
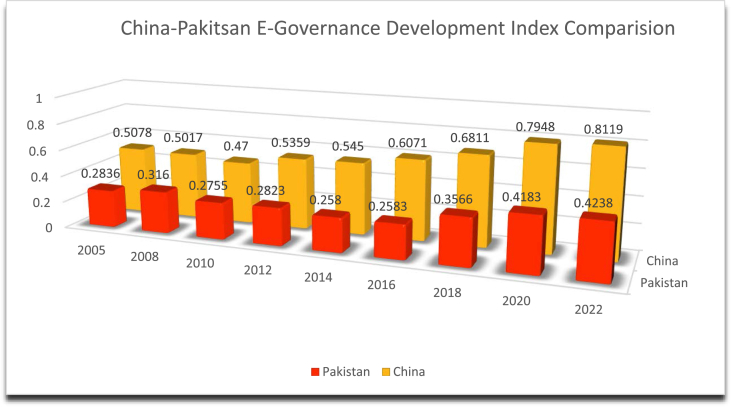
China-Pakistan E-governance development index comparison.

Source: UN E-Government Knowledge Base https://publicadministration.un.org/egovkb/en-us/About/Overview/E-Participation-Index.

China has made rapid development toward E-governance for the last two decades and has become a major player in the area. In China, the mean download speed over mobile and e-government ranking has seen an improvement from 57, to 67 in 2004 [74]. Pakistan's e-government ranking, on the other hand, declined and remained lower as compared to China's ranking. The EDGI of Pakistan saw improvement from 137 in 2003 to 122 in 2004 but then declined steadily [75]. In the year 2008, Pakistan's ranking remains 148/193. In the same year, Pakistan's EDGI was even lower than Bangladesh's and India's. Fig. 1 Based on the EGDI components (Fig. 1), Pakistan needs a better and more modernized e-government model in terms of management, facilitation, and participation and ensures better online services, human development, and telecommunication infrastructure to contribute to the country's development by facilitating e-governance.

A comparison of China-Pakistan key indicators reveals that the overall sustainability and development determinants of Pakistan have seen fluctuations over time. The China-Pakistan graphical comparison of Pakistan in terms of EGDI, HDI, real GDP growth, gross national savings, value-added services, ICT exports, and the Globalization Index (composite of the social, economic, and political indices) shows that China has made significant strides in the field of governance and public administration and e-governance in China has seen drastic improvements. Pakistan's socioeconomic indicators, on the other hand, show that the government needs to improve to take pragmatic steps to improve e-governance ranking.

China has consistently scored higher than Pakistan on the EGDI since 2005. In 2005, Pakistan's EGDI was 0.2836, while China's was 0.5078. By 2022, Pakistan's EGDI had increased to 0.4238, while China's had increased to 0.8119. Both countries showed an overall improvement in their EGDI scores over time. However, China's progress is more remarkable, as it increased its score by a larger margin than Pakistan. In 2005, China's score was more than double Pakistan's score, and this gap continued to widen in subsequent years. Almost all projections of data on E-government and digitalization have shown that China is much ahead of Pakistan. China has performed better due to various reasons. One of the reasons that has been identified is that China has invested heavily in its e-government infrastructure, including online service delivery, telecommunication infrastructure, and human capacity, which has resulted in a significant improvement in its EGDI score. Pakistan's progress has been comparatively slower, although its EGDI has increased steadily over the years. See Fig. 1.

**Fig. 2:**
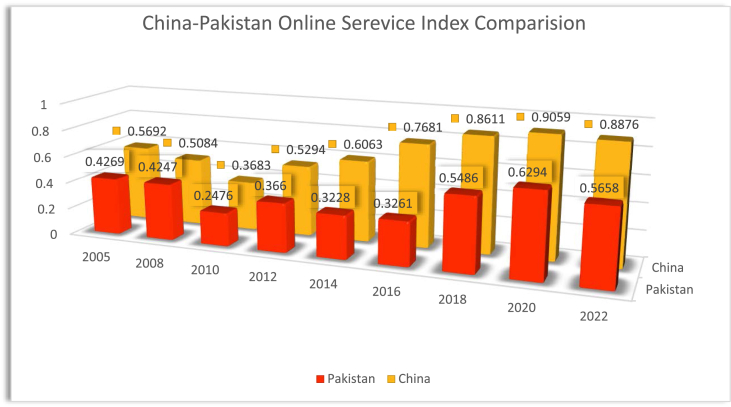
China-Pakistan online serevice index comparison.

Source: UN E-Government Knowledge Base https://publicadministration.un.org/egovkb/en-us/About/Overview/E-Participation-Index.

Based on the EGDI components (Fig. 1), Pakistan needs to implement an effective mechanism of e-government to bring improvement in management, facilitation, and participation. The upgraded system should have the potential to ensure better online services, human development, and telecommunication infrastructure to facilitate e-governance. See Fig. 2.

China has consistently scored higher than Pakistan on the EPI since 2005. In 2005, Pakistan's EPI was 0.127, while China's was 0.1905. By 2022, Pakistan's EPI had decreased to 0.3636, while China's had decreased to 0.8636. In the initial years, Pakistan's EPI was lower than China's, but the trend reversed in 2014 when Pakistan's EPI suddenly jumped to 0.3333, while China's remained stable. Pakistan continued to improve its EPI score until 2020 when it reached 0.5238, but it decreased again in 2022. China, on the other hand, showed steady progress in EPI over the years, with a significant increase between 2014 and 2016 when it jumped from 0.6471 to 0.8136. By 2022, China's EPI had decreased, but it remained significantly higher than Pakistan's EPI. The data for this study has been taken from the UN E-Government knowledgebase (https://publicadministration.un.org/egovkb/en-us/Data/Country-Information/id/128-Pakistan).

Factors contributing to the decrease in EPI in Pakistan include limited information communication technology infrastructure including internet connectivity and electricity issues, especially in rural areas. Digital Literacy; the rural population has low-level skills to use online services. Language barriers; most of the government websites and mobile applications are in English language therefore most of the citizens cannot speak English in resulting limiting the use of digital services. Limited access to technology; high-cost technology and low-income households in developing countries cannot afford to benefit from technology. In rural areas, the digital divide is high in Pakistan. Security Concerns; People may be discouraged from using e-government services if they have real or perceived worries about the security of their personal information and transactions. Establishing confidence in the safety and privacy of digital platforms is essential to their general adoption. Bureaucratic Resistance; The integration of digital technologies into government processes may be hindered by bureaucrats' lack of incentive to adopt E-Government practices and their resistance to change inside government organizations. Policy and Regulatory Issues; Inadequate or outdated policies and regulations may not support the development and implementation of E-Government initiatives. Political Instability and Lack of User-Centric Design (UN 2022 E-Government Survey [26].

**Figure: 3:**
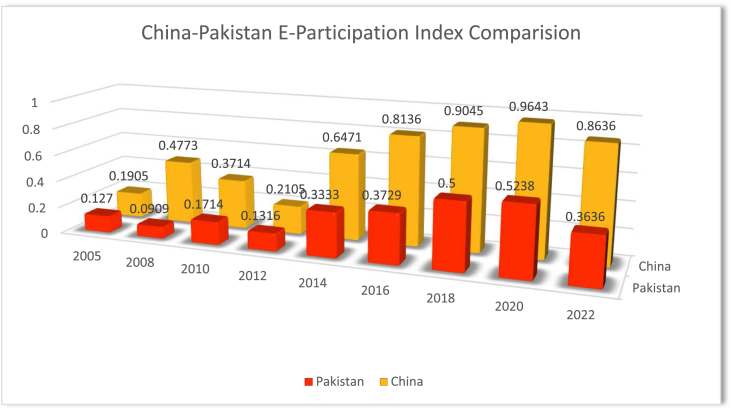
China-Pakistan E-participation index comparison.

Source: UN E-Government Knowledge Base https://publicadministration.un.org/egovkb/en-us/About/Overview/E-Participation-Index.

China has made more progress in increasing citizen participation in e-government services compared to Pakistan. China's overall EPI score remained higher than Pakistan's throughout the years, indicating that China's investment in e-government infrastructure (advanced technology backbone, high speed 5G internet accessibility); Digital Literacy program (Nationwide digital literacy program, integration into the education system); Multilingual Platforms (Implementation of diverse language interfaces, Catering to linguistic diversity); Government Investment Plans (Financial support for E-Government programs, Funding technological advancements); User-Friendly Platforms (Emphasis on intuitive design, Continuous improvement based on user feedback); Integration of Emerging Technologies (Incorporating artificial intelligence and blockchain, Timely enhancing efficiency and security); Public Awareness Campaigns (Informing citizens about E- Government benefits, Promoting active participation); Government Commitment (Strong political support for E-Government initiatives, Ensuring consistency and continuity). Moreover, Cross-Departmental Collaboration (Breaking down silos for seamless integration, Enhancing interdepartmental cooperation); Data Security Measures (Stringent measures to protect citizen data, Building trust through secure platforms); Responsive Customer Support (Establishing effective support system, addressing user queries promptly); Global Best Practices (Learning from successful E-Government models worldwide, implementing proven strategies) and citizen participation programs has been more effective. See Fig. 3.

China-Pakistan comparison in terms of key indicators shows that Pakistan's overall sustainability and development determinants also fluctuate with time. In terms of EGDI, HDI, real GDP, gross national savings, value-added services, ICT exports, and globalization index, show that with proper utilization and implementation e-governance in China has seen significant improvements.

### 4.3 UN policy recommendations for successful adoption of E-government

The United Nations emphasizes several key recommendations to assist the implementation of E-Government. These include the creation of technical infrastructure, the implementation of digital literacy programs, Financial Support and Regulatory Framework, Public-Private Partnerships, Data Security Protocols, demonstrating political will and commitment, Tailoring UN Recommendations to National Contexts, encouraging international collaboration, Overcoming Implementation Challenges, Continuous Monitoring and Evaluation, and the localization of languages to accommodate varied linguistic communities. Overcoming these obstacles is critical to realizing the full promise of digital government. UN Policy Recommendations serve as a guiding framework for E-Government adoption in Pakistan and China. By implementing these recommendations, both nations can overcome challenges, enhance governance, and provide improved public services.

The importance of studying the role of e-governance in promoting good governance cannot be overstated, especially in developing countries such as Pakistan. However, implementing such endeavors can be challenging without a suitable and efficient model to follow. Fortunately, China has shown itself to be a successful example of e-governance implementation, providing valuable lessons for countries like Pakistan.

Effective e-governance can greatly enhance the government's control and involvement in public affairs, promote transparency, and minimize corruption. Additionally, it offers convenient and efficient services to citizens, contributing to overall GDP growth. In the context of the China-Pakistan Economic Corridor (CPEC), e-governance plays an even more critical role, helping to address social and economic problems and facilitating sustainable development. CPEC is an important area and place where Pakistan can implement the Chinese model of E-governance and China is the major partner in this project. However, it is imperative to make advancements in structural, institutional, and policy framework changes in the existing system which is unable to deliver.

It has been reported that Pakistan has different loopholes in its public policies; the same is the case with Digital Policy. To fully realize the potential of e-governance, a reformation of Pakistan's National Digital Pakistan Policy is necessary. This policy must aim to bridge regional digital disparities, streamline procurement and payment systems, and increase access to education and health facilities in remote and underprivileged areas using the Internet. By doing so, Pakistan can achieve progressive transformation across all sectors, ultimately leading to a knowledge-based economy. Without setting rational, science-based, realistic, and concrete policy actions, the implementation of such policies always leads to failure side. There are many other challenges as well that may cause hurdles in effective policy implementation.

Many developing nations are faced with the issue of inadequate resources. They are not able to impose tax payments or transparent systems. The scarcity of financial resources is one of the major reasons for policy implementation failure. In addition, the government collects less tax revenue due to corruption which also results in less confidence among the public. In many developing countries, implementing a modernized e-government system has become a priority. However, they do not have reasonable, human, and technological resources to come up with a pragmatic approach to ensuring effective e-governance.

Transparency is an important aspect of good governance that ICTs can play to ensure it in governmental systems. Countries around the world aim to enhance transparency to build trust by reducing corruption by enhancing online tax filing and bringing improvement in the processing system. Improving the tax system of a country is key to development [76,77]. Examining the role of e-governance in good governance is crucial, especially for developing countries such as Pakistan. Considering the efficiency of China in e-governance and utilization of ITCs in its governance system made a strong case to follow it. Implementing China's model can help any country to modernize its tax-collecting process and improve its governance. Therefore, studying and applying the Chinese case, especially of E-governance for Pakistan is highly relevant where Pakistan can fully revolutionize its fractured and weak governance system.

The last few years have witnessed that China has undergone significant digital transformation. China is considered a leading player in the global digital landscape of the present era. It has initiated various policies, plans, and programs to promote digital transformation which also include the “Made in China 2025" strategy and the “Internet Plus” action plan which are aimed at integrating the Internet and advanced technologies into traditional industries to bring improvement and efficiency. Currently, China is pursuing its plans to develop a global digital superhighway that runs via Pakistan on the advent of CPEC. This “Digital Silk Road” involves laying fiber optic cables in Pakistan which connect with China to the north and link with Africa and the Arab World via undersea cable to be laid from Gwadar Deep Sea Port built as part of CPEC.

The presence of China in Pakistan and digital connectivity is a means to promote and facilitate regional economic cooperation and ensure many ICT integration services between China and Pakistan. This connectivity stretches from many soft to hard infrastructural projects such as paperless trade facilitation, e-commerce, and e-government. These initiatives and cooperation mechanisms are instrumental in the construction and management of industrial parks, roads, rail, aviation, and ports.

## 5 Conclusion

This study investigated the e-governance system in China and Pakistan and made a comparative analysis to show how an effective e-governance system could be implemented in the country and how good governance could be ensured through e-governance. The rationale of the study was how Pakistan can learn from China and adopt China's model of e-governance in the country. For research objectives, we analyzed EDGI, the E-Participation Index, online services, and economic and social measures taken by China and Pakistan. The findings of our research show that China has significantly improved its ranking, whereas Pakistan's ranking has indicated a gradual decline except for the year 2008. Moreover, the study has also identified major challenges for Pakistan's weak standing in e-governance. The major identified reasons include lack of infrastructure, scarcity of financial resources, weak institutional capabilities, and limited access to advanced technologies. Our findings reveal that improving e-government infrastructure is important for good governance. The comparison between China and Pakistan's EGDI and EPI scores over time indicates that effective government policies and investment in e-government infrastructure are critical to achieving sustainable development through e-governance. While both countries have shown progress in their scores, China has made more significant strides in improving its e-government infrastructure, resulting in a higher overall score. Considering the Chinese achievements, Pakistan should adopt a better e-government model to bring improvement in online services ranking, human development progress, and sustainable development through good governance. Investing in e-government infrastructure to promote sustainable development and improve socio-economic indicators has been identified as another key aspect for Pakistan.

This comparative analysis and identification of the remarkable achievements of China in e-governance suggest that Pakistan can learn from China's e-government policies for good governance. E-governance can bring positive and key changes including government control, citizen engagement, transparency, and convenience, while minimizing corruption and boosting GDP growth. Given the growing importance of technology in all sectors, the NDPP must be revised to ensure progressive transformation, boost digitalization, and foster a knowledge-based economy. It is also suggested that effective mechanisms for reducing digital disparities and expanding access to education and healthcare in underprivileged areas through the Internet can integrate the rural and marginalized population in economic development and uplift the poor segment of society. In viewing the China model, Pakistan should have to bring e-government services in a range of areas such as in the economic sector through e-commerce, e-health, e-agriculture, and e-energy etc. The analysis of this research, therefore, suggests that Pakistan needs to implement a value-added digital policy for good governance in the country through e-governance. For this, a digital mechanism can be utilized in the public sector to ensure online services and facilitations. China's e-government achievements are illustrating how e-governance can promote good governance. Pakistan should learn from China and seek collaboration from China for digitalization in the country and bridging the digital divide between rural and urban areas which would help in ensuring good governance in the country.

## Data availability

Data will be made available on reasonable request.

## CRediT authorship contribution statement

**Muhammad Atique:** Writing – original draft, Visualization, Software, Resources, Project administration, Methodology, Investigation, Funding acquisition, Formal analysis, Data curation, Conceptualization. **Su Su Htay:** Writing – original draft, Visualization, Validation, Software, Methodology, Formal analysis, Data curation. **Muhammad Mumtaz:** Writing – original draft, Visualization, Validation, Supervision, Software, Methodology, Investigation, Conceptualization. **Naqib Ullah Khan:** Writing – original draft, Validation, Supervision, Resources, Methodology, Investigation, Formal analysis, Data curation, Conceptualization. **Ali Altalbe:** Writing – review & editing, Resources, Methodology, Funding acquisition.

## Declaration of competing interest

The authors declare that they have no known competing financial interests or personal relationships that could have appeared to influence the work reported in this paper.

## References

[1] Guo H., Wang L., Chen F., Liang D. (2014). Scientific big data and digital earth. Chin. Sci. Bull..

[2] Momen M.N., Ferdous J. (2023). Governance in Bangladesh: Innovations in Delivery of Public Service.

[3] Habib N., Naveed S., Mumtaz M., Sultana R., Akhtar S. (2023). What type of leadership is more effective for managing change during force majeure? Achieving organizational effectiveness during the pandemic. RAUSP Manag. J..

[4] Aritonang D.M. (2017). The impact of e-government system on public service quality in Indonesia. Eur. Scientific J., ESJ.

[5] Guida J., Crow M. (2009). ICT4D: Information and Communication Technology for Development.

[6] Oye N.D. (2013). Reducing corruption in African developing countries: the relevance of E-Governance. Greener J. Soc. Sci..

[7] Srivastava S.K., Panigrahi P.K. (2016). The impact of e-government and e-business on economic performance: a comparative study of developing and developed countries. J. Contemp. Issues Business Govern..

[8] Wu J., Guo S., Huang H., Liu W., Xiang Y. (2018). Information and communications technologies for sustainable development goals: state-of-the-art, needs and perspectives. IEEE Comm. Surveys Tutorials.

[9] Tripathi M., Inani S.K.J. (2020). Does information and communications technology affect economic growth? Empirical evidence from SAARC countries.

[10] Rather R.A. (2020). Customer experience and engagement in tourism destinations: the experiential marketing perspective. J. Trav. Tourism Market..

[11] Kalsi N.S., Kiran R. (2015). A strategic framework for good governance through e-governance optimization: a case study of Punjab in India. Program.

[12] Dwivedi Y.K., Hughes D.L., Coombs C., Constantiou I., Duan Y., Edwards J.S., Gupta B., Lal B., Misra S., Prashant P. (2020). Impact of COVID-19 pandemic on information management research and practice: transforming education, work and life. Int. J. Inf. Manag..

[13] Ullah A., Pinglu C., Ullah S., Abbas H.S.M., Khan S. (2021). The role of e-governance in combating COVID-19 and promoting sustainable development: a comparative study of China and Pakistan. Chinese Polit. Sci. Rev..

[14] Ata-Agboni J.U., Olufemi I.O. (2021). E-governance and e-government: Rethinking public governance in Nigeria, within the context of COVID-19. J. Good Gov. Sustain. Dev. Afr..

[15] Park H., Choi S.O. (2019). Digital innovation adoption and its economic impact focused on path analysis at national level. J. Open Innov. Technol. Market Complex..

[16] Massey G. (2015).

[17] Albadri R.F., Alsallal M.D., Abdulsatar A.S., Abbas Q.H. (2020). The readiness of E-government adoption in Iraq: a case study of Al-muthanna province. Int. J. Psychosoc. Rehabil..

[18] Hasan S., Ullah S.M.A. (2022).

[19] Heeks R., Rakesh V., Sengupta R., Chattapadhyay S., Foster C. (2021). Datafication, value and power in developing countries: big data in two Indian public service organizations. Dev. Pol. Rev..

[20] Akpan-Obong P.I., Trinh M.P., Ayo C.K., Oni A. (2023). E-Governance as good governance? evidence from 15 West African countries. Inf. Technol. Dev..

[21] Suri P.K. (2022). Effectiveness of strategy implementation and e-governance performance. Eval. Progr. Plann..

[22] Lan L. E-government (2004).

[23] Siar S.V. (2005). E-governance at the local government level in the Philippines: an assessment of city government websites. Philippine J. Develop..

[24] Lee-Geiller S., Lee T.D. (2019). Using government websites to enhance democratic E-governance: a conceptual model for evaluation. Govern. Inf. Q..

[25] Banerjee A., Duflo E., Imbert C., Mathew S., Pande R. (2020). E-governance, accountability, and leakage in public programs: experimental evidence from a financial management reform in India. Am. Econ. J. Appl. Econ..

[26] Kim K. (2020).

[27] Bhuiyan S.H. (2011). Modernizing Bangladesh public administration through e-governance: benefits and challenges. Govern. Inf. Q..

[28] Jun K.-N., Wang F., Wang D. (2014). E-government use and perceived government transparency and service capacity: evidence from a Chinese local government. Publ. Perform. Manag. Rev..

[29] Adam I., Fazekas M. (2021). Are emerging technologies helping win the fight against corruption? A review of the state of evidence. Inf. Econ. Pol..

[30] Kim C.-K. (2014). Anti-corruption initiatives and e-government: a cross-national study. Publ. Organ. Rev..

[31] Iqbal M.S., Seo J.-W. (2008). E-governance as an anti corruption tool: Korean cases. 한국지역정보화학회지.

[32] Neupane A., Soar J., Vaidya K., Yong J. (2012).

[33] Akpan-Obong P.I., Ayo C., Adebiyi A. (2016).

[34] Bartenberger M., Grubmüller V. (2014).

[35] Kneuer M., Harnisch S. (2016). Diffusion of e‐government and e‐participation in Democracies and Autocracies. Glob. Policy.

[36] Oseni G., Corral P., Goldstein M., Winters P. (2015). Explaining gender differentials in agricultural production in Nigeria. Agric. Econ..

[37] Masiero S. (2015). Redesigning the Indian food security system through e-governance: the case of Kerala. World Dev..

[38] Ansari, M.M. and J.S. Manhas, Role of ICT in Agriculture.

[39] Gurung S., Dangol S., Bhatta G.P. (2015).

[40] Xia S. (2017). E-governance and political modernization: an empirical study based on Asia from 2003 to 2014. Adm. Sci..

[41] Rubasundram G.A., Rasiah R. (2019). Corruption and good governance. J. Southeast Asian Econ..

[42] Islam S., Dey L.R., Shahriar M., Dewan I., Islam S.A. (2013). Enhancement of dissolution rate of gliclazide using solid dispersions: characterization and dissolution rate comparison. Bangladesh Pharmaceut. J..

[43] Qureshi H.A., Salman Y., Irfan S., Jabeen N. (2017). A systematic review of e-government evaluation. Pakistan Econ. Soc. Rev..

[44] Nishat S., E-Government (2022).

[45] Linders D., Liao C.Z.-P., Wang C.-M. (2018). Proactive e-Governance: flipping the service delivery model from pull to push in Taiwan. Govern. Inf. Q..

[46] Chen A.J., Pan S.L., Zhang J., Huang W.W., Zhu S. (2009). Managing e-government implementation in China: a process perspective. Inf. Manag..

[47] Ma L., Chung J., Thorson S. (2005). E-government in China: bringing economic development through administrative reform. Govern. Inf. Q..

[48] Wang H., Zhang M., Zhong M. (2019). Opportunities and challenges for the construction of a smart city geo-spatial framework in a small urban area in central China. Smart Cities.

[49] Zhou L., Ying M., Wu J. (2021). Conceptualising China's approach to ‘Internet Plus Government Services’: a content analysis of government working plans. Inf. Dev..

[50] Granier B., Kudo H. (2016). How are citizens involved in smart cities? Analysing citizen participation in Japanese''Smart Communities'’. Inf. Polity.

[51] Liu T., Yang X., Zheng Y. (2020). Understanding the evolution of public–private partnerships in Chinese e-government: four stages of development. Asia Pacific J. Pub. Administr..

[52] Norris D.F., Reddick C.G. (2013). Local e‐government in the United States: transformation or incremental change?. Publ. Adm. Rev..

[53] Vyas L., Wu A.M. (2020). Anti-corruption policy: China's Tiger Hunt and India's demonetization. Int. J. Publ. Adm..

[54] Reddick C.G., Zheng Y. (2017). Determinants of citizens' mobile apps future use in Chinese local governments: an analysis of survey data. Transforming Gov. People, Process Policy.

[55] Batool S., Gill S.A., Javaid S., Khan A.J. (2021). Good governance via E-Governance: moving towards digitalization for a digital economy. Rev. Appl. Manag. Soc. Sci..

[56] Alam S. (2013). Successful organization change at national database and registration authority (NADRA) Pakistan: a case study. Global Manag. J. Acad. Corp Stud..

[57] Laila U., Sadiq N., Mehmood T., Fiaz M.F. (2020). E-Governance as a roadmap to good governance: a digital Punjab perspective. J. Account. Finance Emerg. Econ..

[58] Naseem N. (2020). Geopolitical value of gwader for the region (mainly for Pakistan, China and the region). S. Asian Stud..

[59] Khan N.U., Zhongyi P., Ullah A., Mumtaz M. (2024). A comprehensive evaluation of sustainable mineral resources governance in Pakistan: an analysis of challenges and reforms. Resour. Pol..

[60] Hasni M.J.S., Farah M.F., Adeel I. (2021). The technology acceptance model revisited: empirical evidence from the tourism industry in Pakistan. J. Tourism Futur..

[61] Khan N.U., Zhongyi P., Han H., Ariza-Montes A. (2023). Linking public leadership and public project success: the mediating role of team building. Humanit. Soc. Sci. Commun..

[62] Butt N., Warraich N.F., Tahira M. (2019). Development level of electronic government services: an empirical study of e-government websites in Pakistan. Glob. Knowl. Memory Commun..

[63] Rahman M.A. (2018).

[64] Khan N.U., Zada M., Estay C. (2023). Servant leadership and employee prosocial rule-breaking: the underlying effects of psychological safety and compassion at work. PLoS One.

[65] Okeleke K. (2019).

[66] Sumra K.B., Mumtaz M., Mohamed N.D., Haseeb A., Ansari S.H. (2022).

[67] Mumtaz M. (2021). COVID-19 and mental health challenges in Pakistan. Int. J. Soc. Psychiatr..

[68] Mansoor S., Afzal M.T. (2021). Role of TeleSchool in students' engagement during COVID-19 lockdown: a phenomenological perspective. J. Educ. Res..

[69] Bano N., Ali M.A.D.A., Haleemi M.R., Khalid S. (2022). Digitization of Pakistan and PTI government: an analysis. J. Positive School Psychol..

[70] Batool S., Gill S.A., Javaid S., Khan A.J. (2021). Good governance via E-Governance: moving towards digitalization for a digital economy. Rev. Appl. Manag. Soc. Sci..

[71] Mumtaz M., de Oliveira J.A.P. (2023). A framework for analyzing the implementation of climate adaptation policies in the agriculture sector at the subnational level. Environ. Sci. Pol..

[72] Zhong M., Ali M., Faqir K., Begum S., Haider B., Shahzad K., Nosheen N. (2022). China Pakistan economic corridor digital transformation. Front. Psychol..

[73] Ud Din I., Xue M.C., Abdullah, Ali S., Shah T., Ilyas A. (2017). Role of information & communication technology (ICT) and e-governance in health sector of Pakistan: a case study of Peshawar. Cogent Soc. Sci..

[74] Li Y., Shang H. (2020). Service quality, perceived value, and citizens' continuous-use intention regarding e-government: empirical evidence from China. Inf. Manag..

[75] Seo J.-W., Mehedi H., Golam M. (2020). Where are e-governments in South Asian countries? A comparative approach. S. Asian Stud..

[76] Nurunnabi M. (2020).

[77] Shkarlet S., Oliychenko I., Dubyna M., Ditkovska M., Zhovtok V. (2020). Comparative analysis of best practices in e-Government implementation and use of this experience by developing countries. Administratie si Manag. Pub..

